# 3D‐Printed Multi‐Coloured Teeth Comprising Material Gradients for Dental Education—A Pilot Study

**DOI:** 10.1111/eje.13107

**Published:** 2025-05-05

**Authors:** Maximilian Dosch, Falk Schwendicke, Po‐Chun Tseng, Benedikt C Spies, Andreas Keßler

**Affiliations:** ^1^ Department of Conservative Dentistry and Periodontology University Hospital, Ludwig‐Maximilian‐University of Munich Munich Germany; ^2^ Department of Prosthetic Dentistry Medical Center ‐ University of Freiburg, Center for Dental Medicine, Faculty of Medicine, University of Freiburg, Freiburg, Germany. Freiburg Germany

**Keywords:** 3D printing, education, materialgradient, multicolour

## Abstract

**Objective:**

To develop realistic training teeth composed of multi‐material colours and gradients and evaluate them in comparison with the standard model and extracted teeth with a group of students.

**Material and Method:**

Three different teeth were virtually designed by use of multiple STL‐compartments and additively manufactured with different material gradients like colour, hardness and functional properties in a single printing process using MultiJet technology. The teeth included simulated hard‐/softissues and restorative materials like enamel, dentin, pulp, carious dentin, composite, amalgam, and gutta‐percha. The selected teeth were tested by a group of 25 clinical students in a volunteer hands‐on course. They had experience in caries removal, post insertion and preparation on real patients. Study procedures included the removal of a faulty direct and indirect restoration, of gutta‐percha as well as of carious dentin. The properties of the printed teeth for each task were assessed by the students using grades (1 = very good, 2 = good, 3 = satisfactory, 4 = sufficient, 5 = poor). Conventional model teeth and extracted real teeth served as reference.

**Results:**

In comparison to standard model teeth, printed teeth were rated 1.1 ± 0.7, with a grade of 2.4 ± 1.2 for haptic impression and 1.2 ± 0.8 for realistic perception of the exercise. In comparison to extracted teeth, the colour of the enamel(2.1 ± 1.4), the dentin(1.8 ± 1.3) and the carious lesion(1.2 ± 0.8) were evaluated with overall good or very good values. The new features were rated with 1.2 ± 0.7 for the Amalgam filling, 1.0 ± 0.3 for the caries lesion, 1.4 ± 1.5 for the crown, and 2.0 ± 1.0 for the gutta‐percha.

**Conclusion:**

The results of the pilot study confirm the potential of multi‐material additive manufacturing for educational purposes. Students preferred printed teeth in comparison with conventional acrylic and extracted teeth, considering the simulation of a training scenario close to clinical reality.

## 1 Introduction

At the pre‐clinical stage, dental students carry out dental procedures on monocolour simulation models or make use of the extracted teeth. The current gold standard is the preparation of single‐colour, monolithic acrylic teeth, which can only simulate clinical perception to a limited extent, thereby increasing the educational demand at the first clinical application in undergraduate courses treating patients.

At the pre‐clinical stage, dental students usually carry out dental procedures on single‐colour acrylic teeth, which only limitedly reflect on the clinical properties of sound, carious or restored teeth. An alternative to these acrylic teeth is extracted human teeth, while limitations around infection control, their smell, the lack of objectivity and their limited availability are disadvantages [1, 2]. Some companies have therefore launched multicoloured teeth manufactured by injection moulding. Apart from the high costs, however, these teeth can only reproduce the clinically relevant situations (e.g., carious defects) to a limited extent, partly because the manufacturing process cannot comprehensively reflect the complex anatomical structures of sound teeth or the presence of carious lesions or restorations, for instance. The use of models with more realistic properties could lead to better manual and motor skills obtained during preclinical training. In view of increasing restrictions in the treatment of clinical patients, dwindling patient numbers for certain clinical indications and partial restrictions in clinical teaching, for example, during the COVID‐19 pandemic, such training teeth may be highly warranted.

With the advent of innovative manufacturing processes utilising CAD/CAM technology, the production of customised model teeth is now possible in a cost‐effective way. Typical model teeth produced using a 3D printing process with single‐colour resin, based on patient data sets from computer tomography, are available for endodontic, restorative, and surgical procedures, and have been found to enhance learning outcomes [3, 4, 5, 6, 7, 8]. Attempts to create multi‐coloured models that accurately replicate the appearance and function of real teeth have been made with limited success so far [9, 10, 11, 12, 13].

The aim of the present study was to develop and evaluate new multi‐coloured 3D printed teeth with individual material gradients. The simulation of the different compartments within a tooth, including enamel, dentine, pulp, carious dentin, and materials such as gutta‐percha was to be achieved through a single, integrated printing process. By utilising a combination of various printing materials, specifically resin and wax, the final product aimed to provide students with a realistic training experience, closely mimicking the appearance and texture of actual dental tissues.

The developed teeth were to be tested with a group of students and evaluated using a questionnaire.

Our hypothesis was that the 3D printed tooth would have benefits for education in contrast to a standard model tooth in the training of a restorative and prosthodontic situation with filling removal, caries excavation, core build‐up, post insertion, and crown preparation.

## 2 Material and Method

Multi‐coloured printed teeth were created in order to fit into the AG‐3 model (Frasaco, Tettnang, Germany). Specifically, the surface contours of artificial teeth 16, 25, and 35 were digitised using a laboratory scanner (E4, 3Shape, Copenhagen, Denmark). These digitised contours were then superimposed onto the model in the CAD software Blender (Blender Foundation, open source) utilising a 3‐point alignment technique.

**FIGURE 1:**
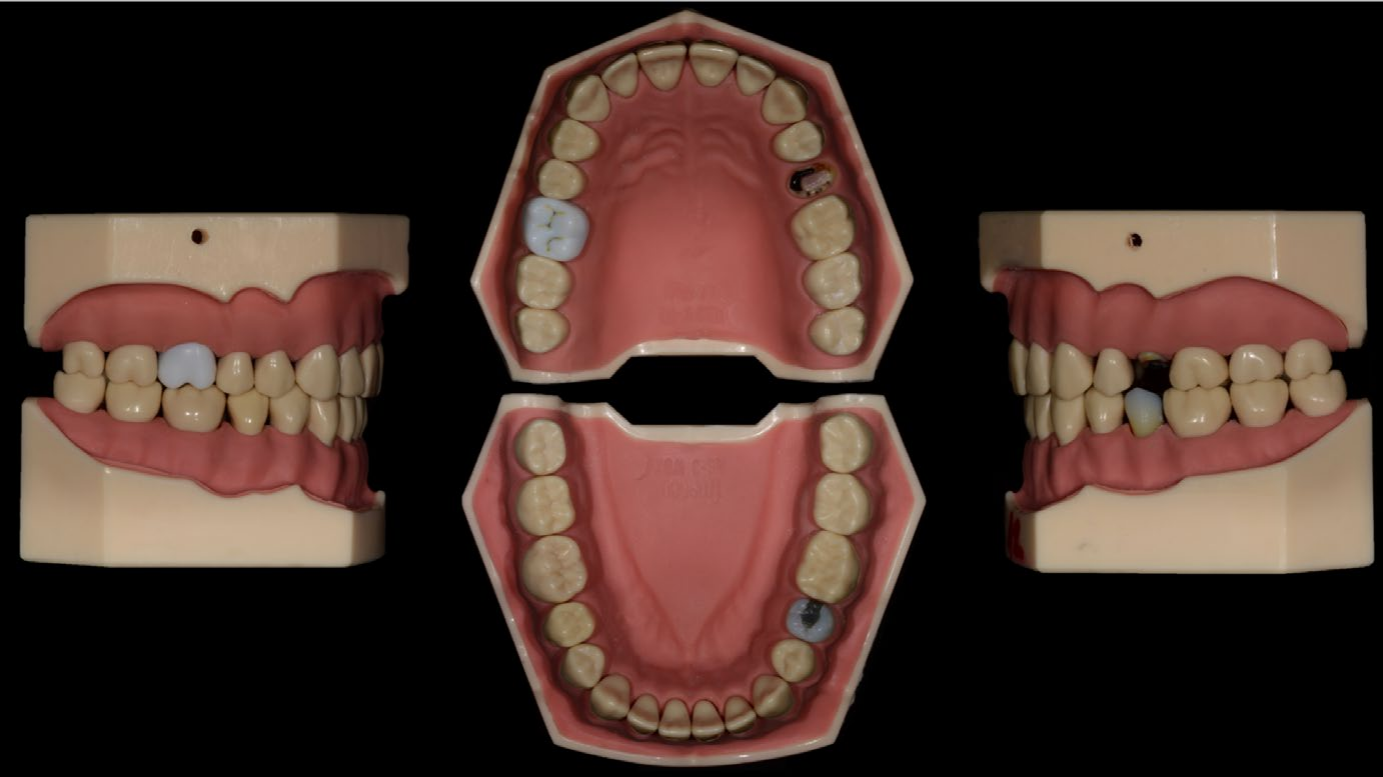
Printed teeth 16, 25 and 35 in the standard model.

Subsequently, the teeth were segmented into various compartments, and defects and special effects were incorporated (Figure 1). Intraoral photographs and the authors' expertise were used for the realistic reconstruction of these compartments. This was achieved by varying the mixing ratios of different printing resins to control the colour, translucency, and hardness of each compartment. The dimensions of these compartments were incrementally optimised, resulting in numerous potential combinations and tooth configurations.

For the study, three exemplary model teeth were selected from the fields of operative and prosthetic dentistry, demonstrating the versatility and precision of the process.

**FIGURE 2:**
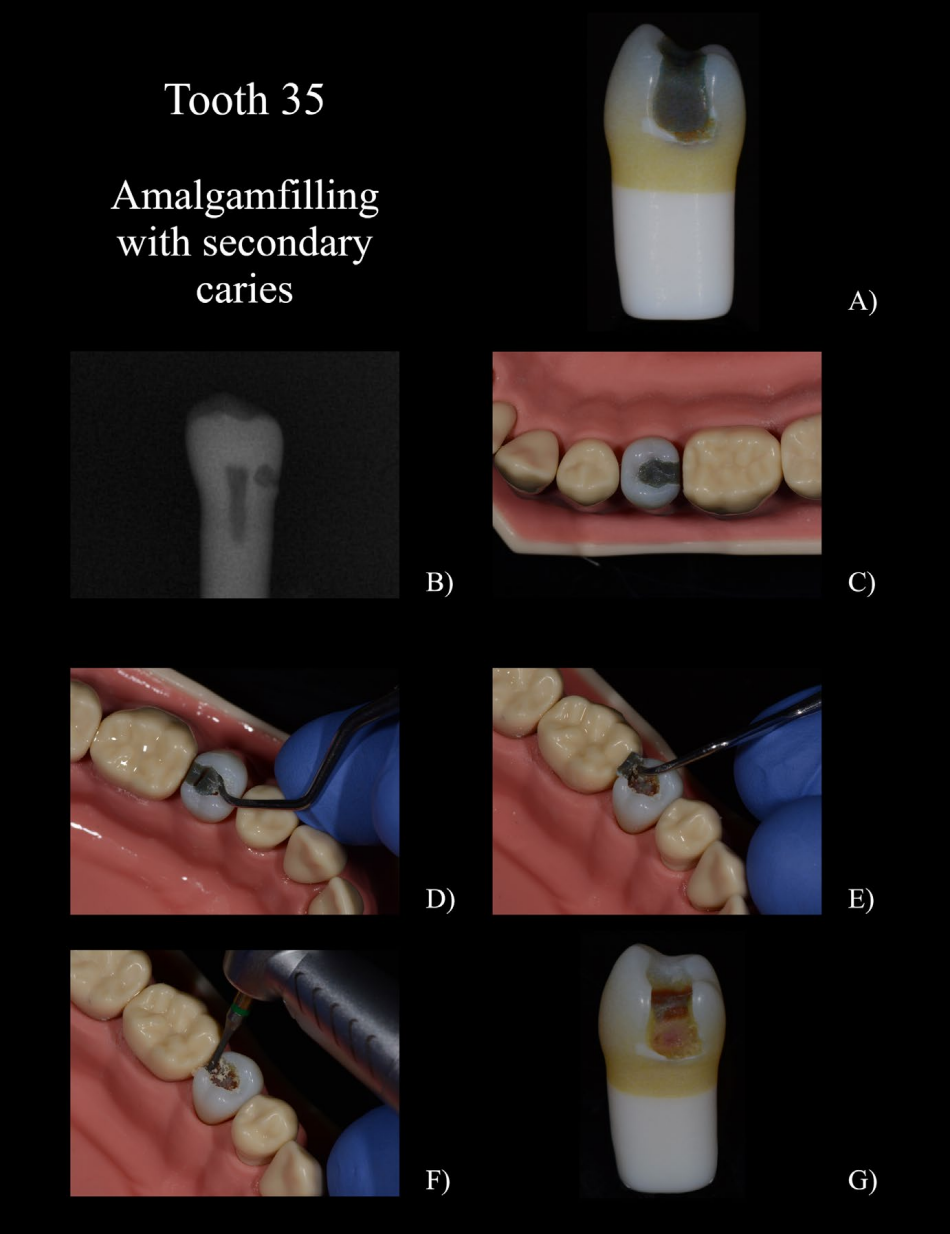
(A) tooth 35 with simulated amalgam filling, enamel opacity and distal secondary caries. (B) X‐ray image with visible caries and pulp. (C) Initial situation from occlusal (D) Separation of the amalgam filling and partial removal. (E) Removal of the distal part using a scaler. (F) Excavation of the caries using a rose bur. (G) Tooth after excavation with visible pulp and discoloured, hard dentin.

**FIGURE 3:**
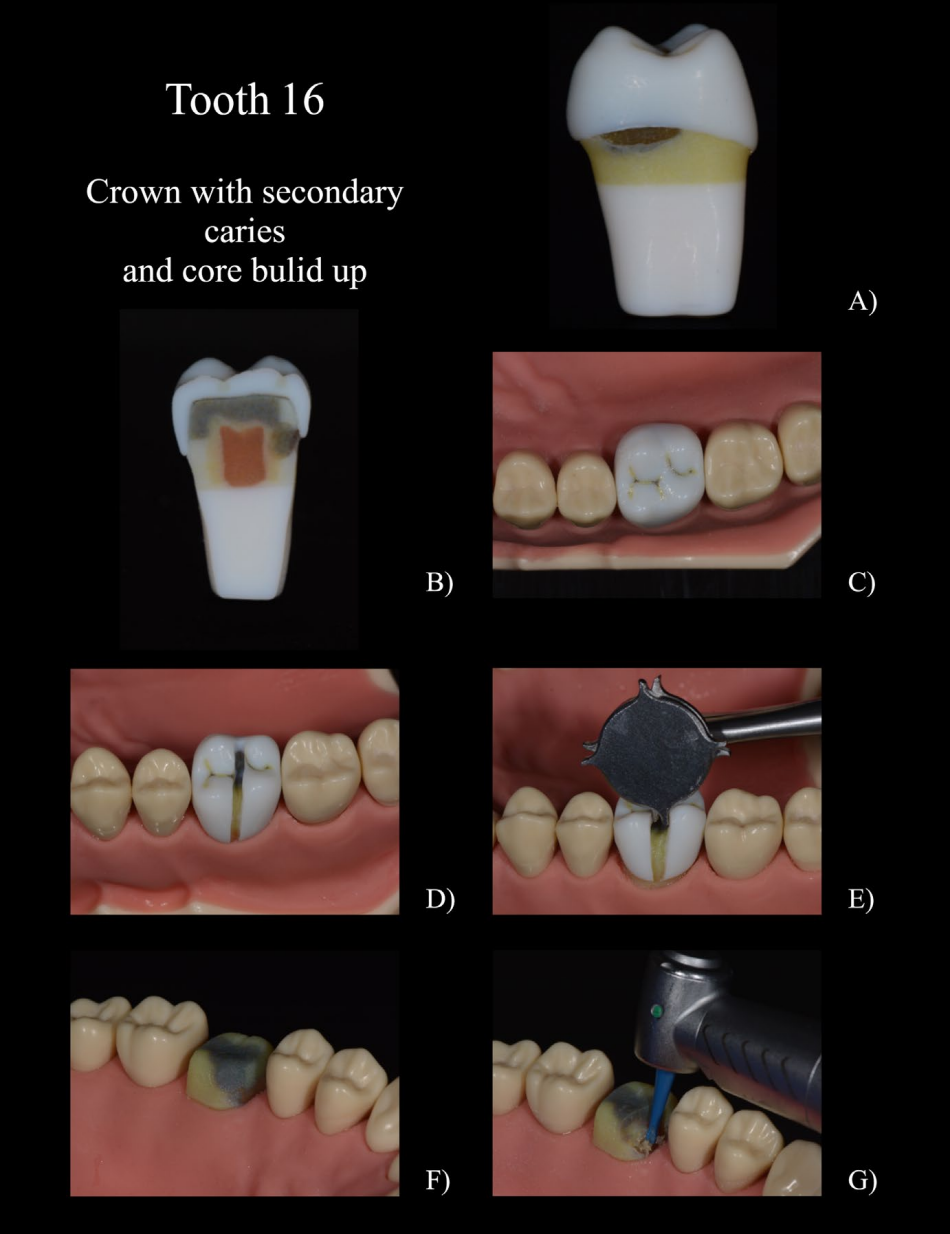
(A) 16 with simulated crown and secondary caries. (B) 16 in cross‐section with the compartments crown, dentin, core build‐up and caries. (C) Crown with occlusal discolouration. (D) Sliced crown (E) Bending up of the crown using a crown spreader. (F) Discoloured core build‐up with mesial caries. (G) Partial removal of the core build‐up and excavation using a polymer drill, for selective caries excavation.

**FIGURE 4:**
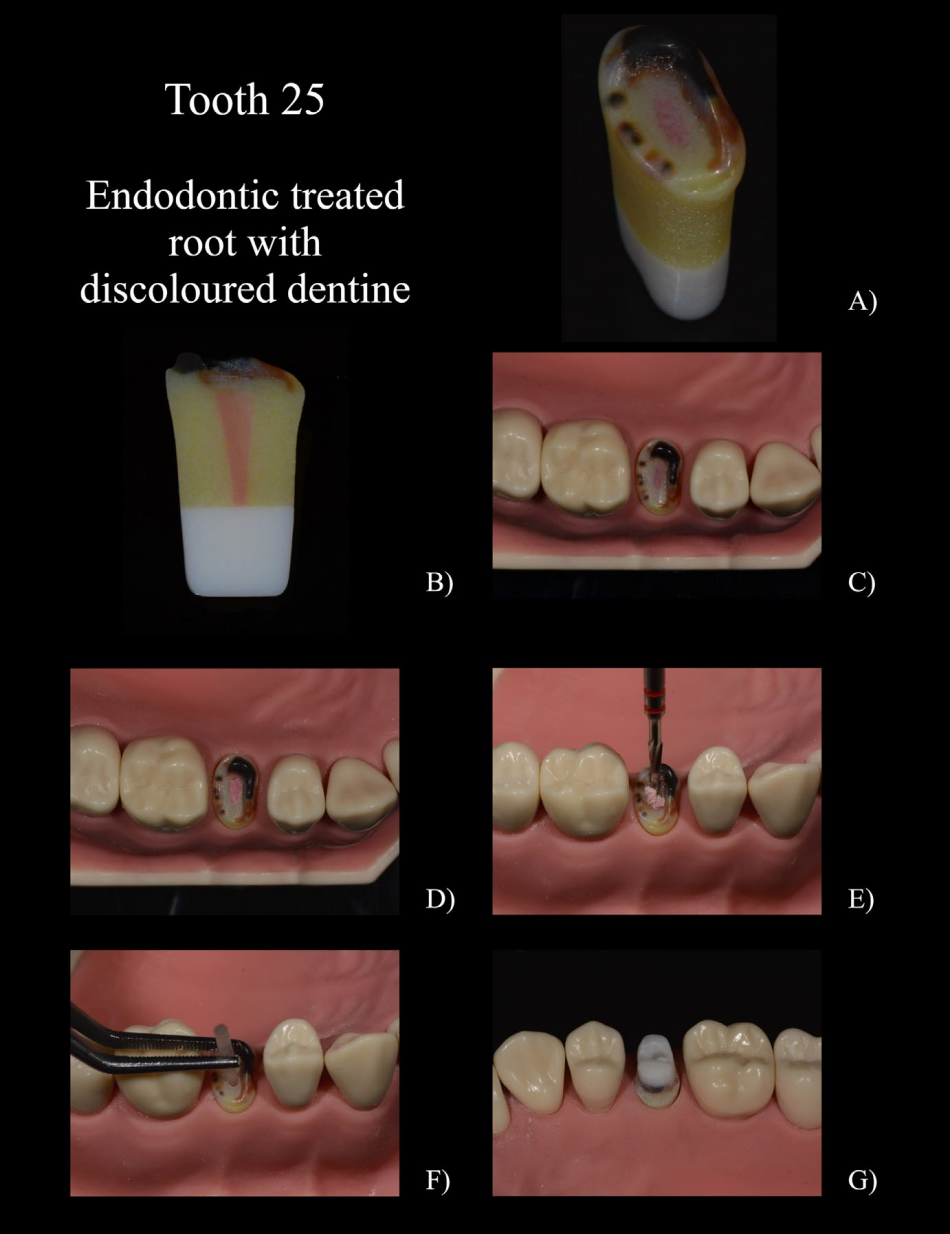
(A) 15 with fractured crown, covered root filling and discoloured, hard dentin. (B) 15 in cross‐section with simulated gutta‐percha. (C) Situation from occlusal. (D) Removed covering filling. (E) Post drilling with simulated gutta‐percha chips. (F) Insertion of a glass fibre post. (G) Preparation of the tooth with core build‐up and discoloured dentin.

1. A filling was designed for tooth 35 (FDI‐Scheme, Figure 2) to simulate the appearance of amalgam. It was specifically designed and printed not to firmly bond to the surrounding structure. This design enabled the filling to be separated and removed, like amalgam in a clinical scenario. In addition, a simulated secondary caries was located at the distocervical margin of the filling; the simulated decayed dentin revealed a softer consistency compared to healthy dentin and extended into the inner dentin layer near the pulp. The dentin's composition allowed for slightly moist cutting removal with a rose bur. While self‐limiting polymer burs (e.g., P1 Polybur, Komet, Lemgo, Germany) were able to remove the carious dentin, they wore out when encountering the harder healthy dentin. There was a seamless transition between the simulated carious and healthy dentin. To enhance realism, colour accents like white opacities on the distal enamel margin were additionally added.
2. A temporary crown including secondary caries at the mesial aspect and a central, adhesively bonded core build‐up (region 16, Figure 3). The simulated cementation of the crown enabled it to be spread during crown removal, facilitating the complete removal of the crown in a realistic manner. The simulated secondary caries extended under both the crown and the build‐up filling (Figure 3).
3. Tooth 25 (Figure 4) simulated a root remnant worth preserving after simulated crown fracture with sufficient root filling, covered by composite. The dentin was coronally discoloured but hard during probing. The simulated gutta‐percha was printed with a softer material, only sticking to the surrounding root, therefore allowing it to be removed using a drill.

The STL files were nested for 3D printing using GrabCAD (Cambridge, Massachusetts, USA) and produced by a Polyjet 3D printer (J850 Digital Anatomy‐printer, Stratasys, Rechovot, Israel) and subjected to post‐processing. Teeth were produced by Hager&Meisinger (Hager&Meisinger, Neuss, Germany).

Criteria for participation of the students were at least one semester of clinical patient treatment and an undergraduate degree. Students with clinical experience were deliberately sought in order to gain a better assessment of the practical suitability of the teeth. A total of 25 students took part in the study (16 women, 9 men) in between the semester holidays. Six of them completed their 7th semester, four their 8th semester, 13 their 9th semester, and two their 10th semester.

The study was conducted at the clinical student course of the Department of Conservative Dentistry and periodontology of the University Hospital of Munich. For this purpose, the fully equipped treatment units were fitted with a phantom head. In addition to the visual and tactile examination, the students were also provided with the radiological image of tooth 35. Afterwards, the students explained and discussed the diagnosis and treatment planning and carried out the treatment of the teeth independently. As in the clinical courses, this was done under the supervision of an assistant dentist, who was available to answer any problems or questions.

After the treatment, all students were given a questionnaire to evaluate their experiences with the experimental teeth. The questionnaire was based on previously published studies and was completed anonymously [3, 4, 10]. The teeth were compared with standard model teeth and extracted human teeth based on the experience of the students. The special features of the experimental teeth were evaluated in addition. The questions were designed as closed questions to be scored on a scale (1 = very good, 2 = good, 3 = satisfactory, 4 = sufficient, 5 = poor) for good discrimination. Finally, a free text question was included in which participants had to rate the educational effect of the 3D printed teeth, real teeth, and standard model teeth. The descriptive analysis was carried out using Excel 23.12 (Microsoft, Redmond, Washington, DC, USA).

## 3 Results

### 3.1 Comparison to a Standard Model Tooth

The haptic impression of the new multi‐coloured printed teeth during preparation was rated with a mean ± SD grade of 2.4 ± 1.2 compared to standard model teeth. However, the new teeth received a grade of 1.2 ± 0.8 when asked if they allowed realistic procedures and were clearly favoured over the mono‐coloured standard model teeth (1.1 ± 0.7).

### 3.2 Comparison to Extracted Human Teeth

**FIGURE 5:**
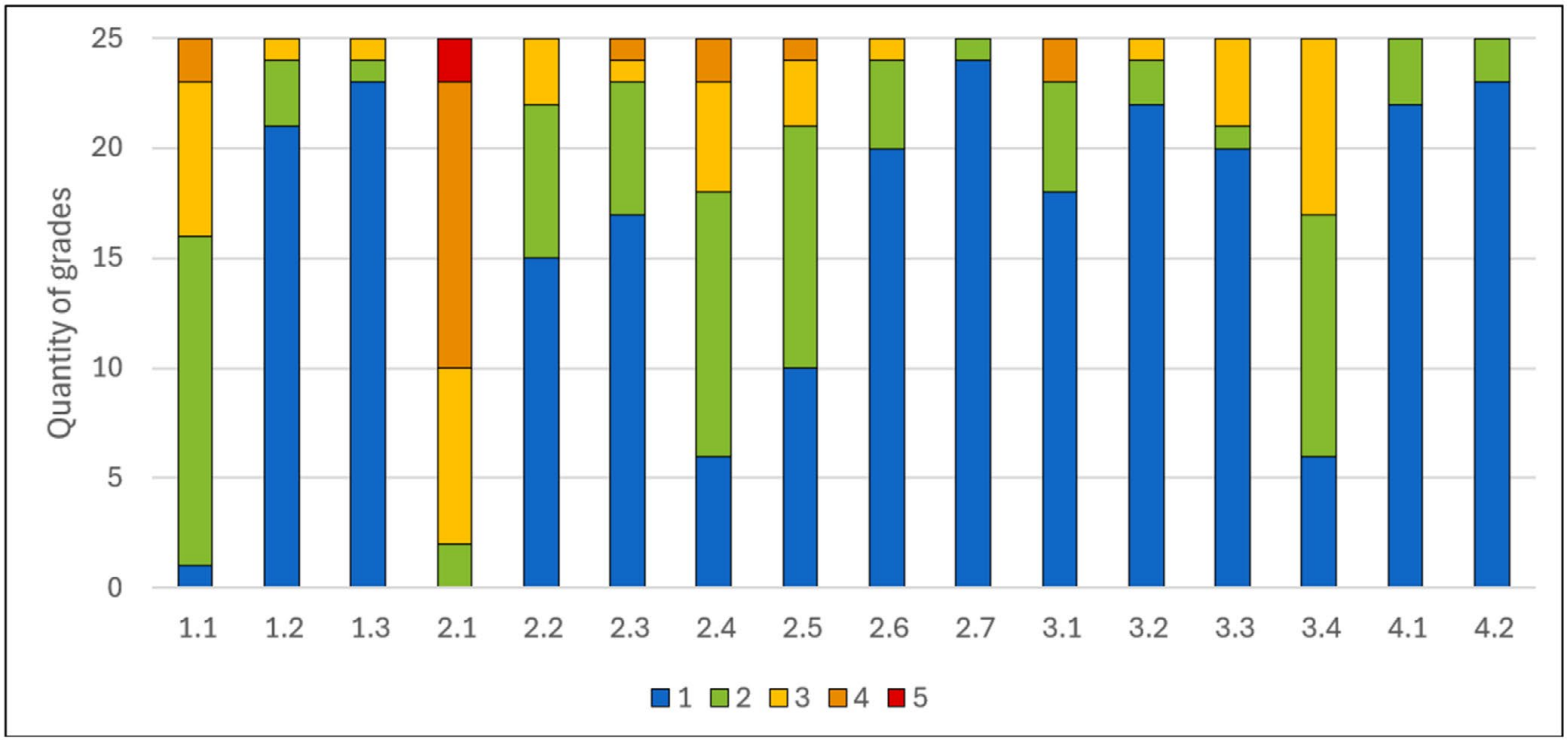
The results of the evaluation were visualised as a bar chart. The questions were designed as closed format rating scale questions divided into school grades (1 = excellent, 2 = good, 3 = adequate, 4 = sufficient, 5 = poor). Items for comparison of the printed tooth to a standard model tooth were as follows: (1.1) haptic impression at preparation (1.2) suitable exercise option (1.3) Preference compared to standard acrylic teeth. Items for comparison of the printed tooth to a real extracted tooth were as follows: (2.1) haptic impression at preparation in enamel (2.2) haptic impression at preparation in dentin (2.3) haptic impression at preparation at caries excavation (2.4) Colour of enamel (2.5) Colour of dentin (2.6) Colour of caries (2.7) Are more suitable than extracted teeth. Items for the evaluation of the new features: (3.1) Removal of the amalgam filling (3.2) Caries excavation (3.3) Removal of the crown (3.4) Gutta‐percha reduction and post preparation. Items for assessment of the learning process with the printed tooth were as follows: (4.1) Could have a positive impact on teaching (4.2) Would better prepare students for clinical courses.

The results of the comparison of the printed with extracted teeth are shown in Figure 5 (2.1–2.7). The new teeth received limitedly good grades (3.6 ± 1.4) for enamel preparation properties. In the opinion of the students, the printed teeth proved to be well suited to reproducing the properties of the dentin during preparation (1.5 ± 1.0) and the carious dentin during excavation (1.4 ± 1.3). The colour of the enamel (2.1 ± 1.4), the dentin (0.8 ± 1.3) and the carious lesion (1.2 ± 0.8) were evaluated with overall good or very good average grades. As expected, all students rated the printed teeth as more pleasant in terms of hygiene compared to the extracted teeth.

The answers in the free text section at the end of the questionnaire provide further details. The simulation of carious dentin was once again emphasised as particularly positive. The main disadvantage mentioned was that there was no clear distinction in hardness between enamel and dentine. The comments in the free text section of the questionnaire also showed that the majority of students found the printed teeth to be realistic and suitable for exercises.

### 3.3 Special Features

In the penultimate part of the questionnaire, the special features of the printed teeth were assessed individually (Figure 5, 3.1–3.4). All printed teeth received good to very good grades. The best result was achieved by the printed tooth with the amalgam filling (1.2 ± 0.7) and the one with secondary caries (1.0 ± 0.3). All students were able to remove the amalgam filling from the cavity walls using a scaler after partial separation of the amalgam filling. The replication of the carious lesions and the possibility of removal using steel and polymer burs was emphasised as particularly realistic in comparison to their natural counterparts. The hard but discoloured dentin after caries excavation was also found to be very realistic. With regard to the radiographic image, many students expressed the wish for a subdivision into enamel and dentin in the free text. Tooth 16 with the simulated crown and secondary caries was graded as 1.4 ± 1.5. The possibility to slot the crown and subsequently split it was particularly well received. The different procedure for removing the simulated adhesive filling versus the amalgam filling in tooth 35 was also positively emphasised. The lowest score was given to tooth 25 with gutta‐percha (2.0 ± 1.0).

## 4 Discussion

Today's treatment for restorative therapy is based on many factors on the part of the patient (caries risk, erosions, parafunctions, residual tooth substance) as well as on the part of the materials used. Clear metric preparation guidelines such as those provided by textbooks or standardised teeth are therefore only of limited help for the students. Individually 3D printed teeth that simulate clinical situations enable a more realistic training of modern defect‐oriented treatment concepts and are useful in acquiring pertinent hands‐on skills for clinical application. The combination of computer‐aided design and 3D printing developed for teaching purposes and assessed by questionnaire proved to be a viable method for various fields of dentistry. Likewise, in our study a questionnaire for the evaluation of the printed tooth model was based on a questionnaire already tested and proven in multiple studies before [3, 4, 10].

A key consideration concerns the material a model is 3D printed from and its basic properties. Monochrome printed teeth using SLA and DLP are most widely used and evaluated to date [5, 7, 8, 13]. Sometimes they were printed in different parts and colours, painted, and finally stacked together [3, 4, 10, 12]. However, this has the disadvantage of additional work; various parts may not contain undercuts, and it can result in adhesive problems, as shown by Sonkaya. Other authors attempt to print multicoloured models using multijet technology [11]. However, these were printed in layers, using abstract, bright colours without material gradients or the function of the individual compartments. Through continuous improvement of the CAD design in this study, in combination with various experiments regarding colour, translucency, and material properties, it was now possible to print study teeth aiming to be comparable to real teeth in terms of their appearance and treatment steps. In comparison to single‐coloured model teeth, the students included in the present study rated the teeth to be more realistic and comparable to extracted teeth. Overall, they preferred them over extracted teeth. Extracted teeth in particular have some disadvantages, as already addressed in previous studies [8, 12, 13]. Extracted teeth must be sterilised to prevent contamination with residues of human tissue. Ultimately, however, the unpleasant smell remains even after sterilisation [1, 2] and availability is limited. Finally, extracted teeth vary in shape and consistency, thereby limiting the comparability of the exercises. Besides ethical considerations, this does not allow for standardised and objective examinations. Consequently, 3D printed teeth offer enhanced hygiene, improved handling, and the possibility of standardising case situations, ensuring a consistent level of difficulty for each student. In the realm of didactics, it holds paramount significance to illustrate to students the subtle nuances in colour between enamel and dentin during the preparation phase. This realistic portrayal proves crucial for guiding subsequent steps in the adhesive technique with precision and efficacy.

Clinically, it is of importance whether a preparation and cavity are located within the enamel or in the dentin. The colour gradation between enamel and dentin was rated as realistic by the students. This means that important steps such as selective enamel etching or veneer preparation can be taught realistically. However, a persistent problem with printed teeth is the hardness of the simulated enamel during preparation, as confirmed by the students. In the future, improvements will have to be made to the material properties. MultiJet printing relies on photopolymer resins, which are typically liquid‐based materials that cure under UV light. Material properties can be enhanced by the use of inorganic fillers. However, inorganic fillers (such as ceramic, silica or hydroxyapatite) are solid particles that are difficult to disperse evenly in the resin without affecting viscosity and printability. Furthermore, MultiJet printers jet liquid resin droplets through tiny nozzles. If the resin contains solid fillers, these nozzles can become clogged, disrupting the printing process. Secondly, the degree of cure can be enhanced by greater exposure to UV light during post‐processing.

A special feature of the newly developed teeth was the simulation of carious dentin, which was rated positively by the students in terms of appearance and hardness on removal. In addition to the shape, size, and localisation of the carious dentin within the tooth, a pseudo‐intact enamel layer or the relationship to a restoration in the sense of secondary caries, as in the study teeth, can also be implemented. The carious dentin consists of a soft, slightly moist, brownish material that can be removed with a rose bur. In our study, the carious dentin was part of a secondary lesion under an amalgam filling, extending close to the pulp. For tooth 16, a defect at the crown margin was simulated to represent secondary caries, extending beneath the core build‐up towards the pulp. Due to the specially adapted hardness, the caries can also be selectively removed (Figure 3G). A layer of discoloured dentin was printed adjacent to the caries to be removed. This dentin, being harder, wears down polymer burs and effectively simulates the smooth transition between different dentin zones (Figure 2G).

A reddish pulp was also simulated, which produced a characteristic image in the dentin in deep cavities due to the anatomical proximity (Figure 2G). Two students performed the excavation with too much pressure and opened the pulp. The pulp is therefore simulated with a red, soft material and enables partial removal. The two students responded very positively to the question about the colour differentiation of the pulp.

Höhne and Sonkaya used radiographs for diagnostic purposes and to guide cavity preparation [3, 12]. In contrast to the two studies mentioned, no different parts were produced for enamel and dentin in our teeth and combined in the second step. As a result, no differences in radiopacity between dentin and enamel can currently be realised, while the pulp and caries have a lower radiopacity due to their softer material in the X‐ray image (Figure 2B). This was criticised by the students in the free text of the questionnaire. However, it was also emphasised that the distinction between carious dentin, pulp, and the enamel‐dentin compartment was clearly visible. Future experiments should demonstrate the possibility of creating a different radiopacity of enamel and dentin by adding X‐ray contrast agents (such as yttrium fluoride).

The transitions between the simulated amalgam filling and the crown restoration to the tooth were designed and printed to allow partial removal of the respective restoration after separating it appropriately (Figure 2D,E). The crown could be expanded with the crown spreader and removed in total (Figure 3D,E). This special feature was rated as very good by the students. Multicolour 3D printing makes it possible to reproduce freeforms in any way, thereby avoiding the need to assemble model teeth from different parts as presented in previous studies.

The results of the last part of the study questionnaire confirmed the hypothesis that multicoloured 3D printed teeth with different material gradients have the potential to improve the formation compared to a standard model tooth. As a result, they have the potential to be used not only for examinations, but also for post‐graduate training if necessary.

One limitation of the study is that it was conducted as a pilot study at a single university and its results are not transferable to all students. The students referred their evaluation on their experience with plastic and real tooth—there was no direct control group. The main disadvantage of the simulated enamel is the low mechanical properties (modulus of elasticity and hardness) compared to natural enamel. The evaluated new teeth are intended to complement rather than replace conventional teaching methods to train specific anatomical or pathological features that cannot be replicated using monocolour acrylic or extracted teeth. Further multicentral studies are planned with different student levels to increase the sample size and technical skills diversity. The objective of these studies is to evaluate the impact of teeth on the student curriculum, in contrast to conventional teeth. The evaluation will be conducted using questionnaires, expert evaluation, and metric analysis of prepared teeth.

## 5 Conclusion

The use of multicoloured 3D printed teeth comprising different material gradients successfully allowed the simulation of tooth anomalies, incorporating essential anatomical and pathological features. The results of this study confirmed the potential of additively manufactured multi‐material teeth, which were rated significantly better by students than standard model teeth or extracted teeth. The experimental teeth are intended to provide the opportunity to prepare for daily work on patients in a more varied and better way and thus lead to a higher quality of clinical treatment later on.

## Conflicts of Interest

The authors declare no conflicts of interest.

## Data Availability

Data available on request from the authors.
